# Risk prediction models for malignant cerebral edema after endovascular therapy in patients with acute anterior circulation large vessel occlusion stroke: a systematic review and meta-analysis

**DOI:** 10.3389/fneur.2026.1686413

**Published:** 2026-02-05

**Authors:** Yuan Yuan, Weixin Cai, Yadi Feng, Ran Zhang, Cuixue Wang, Yifan Luo, Xiaoping Yi

**Affiliations:** 1 Department of Nursing, Beijing Tiantan Hospital, Capital Medical University, Beijing, China; 2 Intensive Care Unit, Beijing Tiantan Hospital, Capital Medical University, Beijing, China; 3 Department of Neurology, Beijing Tiantan Hospital, Capital Medical University, Beijing, China

**Keywords:** endovascular therapy, large vessel occlusion stroke, malignant cerebral edema, prediction model, risk factor, systematic review

## Abstract

**Background:**

Endovascular therapy (EVT) is proven to be both effective and safe for treating acute anterior circulation large vessel occlusion stroke (ACLVOS). Malignant cerebral edema (MCE) can emerge as a severe complication following ET. Predicting acute ACLVOS patients at risk of MCE is crucial for prevention, management, and medical decision-making. The predictive performance and predictive factors of MCE models are not yet well understood.

**Aims:**

To identify risk prediction models and potential predictive factors for malignant cerebral edema (MCE) after endovascular therapy (EVT) in patients with acute anterior circulation large vessel occlusion stroke (ACLVOS).

**Study design:**

We conducted a systematic search of studies using eight databases from inception until December 31st, 2024. Data extraction followed the critical appraisal and data extraction for systematic reviews of prediction modelling studies (CHARMS) Checklist. And We used the prediction model risk of bias assessment tool (PROBAST) tool to assess the risk of bias and applicability.

**Results:**

We included 21 articles identifying 30 MCE prediction models. The most common predictors of MCE were the National Institutes of Health Stroke Scale (NIHSS) score, collateral score, and Alberta Stroke Program Early Computed Tomography Score (ASPECTS). All included studies exhibited a high risk of bias. Seventeen studies raised significant applicability concerns, whereas five studies posed lower applicability concerns.

**Conclusion:**

This systematic review confirms the feasibility of predicting MCE risk after EVT in ACLVOS patients using existing models and highlights key predictive factors. However, the high risk of bias across studies limits their clinical applicability.

**Relevance to clinical practice:**

This study empowers ICU nurses to accurately identify the risk levels of MCE and implement targeted monitoring strategies.

**Systematic review registration:**

https://www.crd.york.ac.uk/PROSPERO/view/CRD42024564544, CRD42024564544.

## 1 Introduction

Stroke is a global health challenge with a rising incidence rate (1) and is ranked as the second leading cause of death worldwide and the primary cause of adult disability (2). Ischemic stroke, the most common type (3), occurs when cerebral blood flow is obstructed, leading to brain tissue damage and poor outcomes (4). Notably, among ischemic stroke’s subtypes, cardioembolic and atherothrombotic strokes are particularly critical, as they are associated with the highest in-hospital mortality (5). Endovascular therapy (EVT) is an effective and safe intervention for acute anterior circulation large vessel occlusion stroke (ACLVOS) (6–8). However, 10–75% of patients develop malignant cerebral edema (MCE) after treatment (9), a severe neurological complication typically occurring within 7 days (10). MCE can cause elevated intracranial pressure, midline shift, cerebral herniation, and even death (11). Early surgical decompression may mitigate MCE-related damage, improving outcomes and survival rates (12), but its benefits are constrained by MCE progression. Consequently, early prediction and clinical prevention strategies are critical to reducing MCE burden (11).

## 2 Background

Numerous predictive models and factors for MCE have been reported. A systematic review identified 44 predictive factors and four models (13), but limitations in study design have hindered their widespread adoption. Several well-established models for ischemic stroke prediction include the Kasner score (14), EDEMA (Enhanced Detection of Edema in Malignant Anterior Circulation Stroke) score (15), and Malignant Brain Edema (MBE) score (16). However, these models lack full external validation in EVT patients. Additional models have been published and validated, incorporating machine learning and novel predictive factors (17). In a hospital-derived clinical dataset, impaired consciousness, nausea or vomiting, and a history of heavy smoking stood out as the main clinical predictors linked to malignant middle cerebral artery infarction (18). Given these advancements, a systematic review is warranted to assess the characteristics, quality, and developmental trends of MCE prediction models.

## 3 Aims and objectives

This study aimed to review prediction models for MCE in ACLVOS patients after EVT. The specific objectives were to (a) identify the predictive factors of each model and (b) evaluate the performance of different models.

## 4 Design and methods

This systematic review adhered to the Preferred Reporting Items for Systematic Reviews and Meta-Analyses (PRISMA) 2020 guidelines (19). The PECOS framework was employed to define the review objectives, structure the search strategy, and establish inclusion and exclusion criteria (20). The PECOS framework included the following components: P: patients with acute anterior circulation large vessel occlusion stroke; E: with malignant brain edema; C: without malignant brain edema; O: risk factors, risk prediction models; S: case–control, or cohort, or RCTs.

### 4.1 Search strategy

Based on a comprehensive literature search, we systematically reviewed both Chinese and English databases. The databases searched included Medline/PubMed, Web of Science, Embase, the Cumulative Index to Nursing and Allied Health Literature (CINAHL), China National Knowledge Infrastructure (CNKI), Wanfang Database, VIP Information, and the Chinese Biomedical Mathematical (SinoMed) Database from their inception until December 31st, 2024. The search strategy incorporated a combination of keywords and free-text terms, including “malignant cerebral edema,” “severe cerebral edema,” “life-threatening edema,” “stroke,” “neurological critical illness,” “cerebral infarction,” “cerebral blood vessels,” “endovascular therapy,” “thrombectomy,” “stenting,” “prediction,” “risk factors,” “models,” “machine learning,” and “ROC curves.” Detailed search strategies and specific queries are provided in Supplementary material A. Additionally, we manually screened the reference lists of retrieved studies and relevant review articles to identify supplementary studies. All retrieved studies were imported into NoteExpress reference management software for organization and subsequent analysis.

### 4.2 Eligibility criteria

Studies were included in this review if they met the following criteria: (1) involved adult stroke patients (aged ≥18 years); (2) focused on MCE with clearly defined diagnostic criteria, including but not limited to radiographic evidence, a midline shift of at least 5 mm, or clinical symptoms indicative of ventricular compression or cerebral herniation; (3) reported at least one prediction model incorporating a minimum of two predictors; and (4) employed an observational study design or secondary analysis of an existing database.

The exclusion criteria were as follows: (1) studies published in languages other than English or Chinese; (2) duplicate publications; (3) studies for which full text and complete data were unavailable despite making attempts to contact the authors; and (4) reviews, congress abstracts, or commentaries.

### 4.3 Study selection

Duplicate studies were removed using NoteExpress (version 4.0). Two authors, both trained in systematic review methodology, independently screened studies based on the inclusion and exclusion criteria. Discrepancies were resolved through discussion with a third author. Initially, titles and abstracts were reviewed, followed by full-text screening. Finally, the reference lists of the included studies were checked to identify additional relevant studies.

### 4.4 Data extraction

Two authors independently extracted data, followed by a cross-check to ensure accuracy. Any disagreements were resolved through consultation with a third author. Data extraction adhered to the CHARMS (Critical Appraisal and Data Extraction for Systematic Reviews of Prediction Modelling Studies) checklist (21). Extracted information included study characteristics and model details. Study characteristics comprised author, year of publication, country, study type, design, participant characteristics, treatment details, prediction timeframe, sample size, diagnostic criteria, and MCE incidence. Model details included the number of selected variables, variable selection methods, handling of continuous variables, model presentation, model development and validation approaches, performance metrics, identified risk predictors, and methods for handling missing data.

Model performance was assessed using discrimination and calibration metrics (22). Discrimination was evaluated through the area under the receiver operating characteristic curve (AUC) or C-index, which measures the model’s ability to distinguish between individuals with and without the outcome. The AUC or C-index values were reported explicitly rather than categorized arbitrarily. Calibration was assessed using calibration plots or the Hosmer–Lemeshow test, which evaluates the agreement between predicted and observed outcomes (23). Additionally, the frequency of predictive factors was analyzed to determine their relative contributions to MCE prediction.

### 4.5 Quality appraisal

The risk of bias in the included studies was assessed using the Prediction Model Risk of Bias Assessment Tool (PROBAST) (24). Two authors, trained in PROBAST methodology, independently evaluated bias risk and applicability concerns. Discrepancies were resolved through discussion with a third author. PROBAST, encompassing 20 specific items, was used to evaluate four domains: participants, predictors, outcomes, and analysis. Each item was rated as “yes,” “probably yes,” “probably no,” “no,” or “no information.” Studies were classified as having a low, high, or unclear risk of bias. If all items within a domain were rated as “yes” or “no information,” then the domain was deemed to have a low or unclear risk of bias. However, if any item was rated as “no” or “probably no,” the domain was classified as having a high risk of bias. A study was considered to have a low overall risk of bias if all four domains were rated as low bias, whereas a high risk of bias in any domain resulted in a high overall risk assessment.

### 4.6 Data synthesis and statistical analysis

Statistical analyses were conducted using Stata (version 16.0). A meta-analysis was performed on AUC values, and subgroup analyses were performed, where applicable. Two stroke physicians independently assessed clinical heterogeneity to determine the appropriateness of conducting a meta-analysis. Statistical heterogeneity was quantified using the *I*^2^ statistic (25). Based on the degree of heterogeneity, studies were pooled using either fixed-effect or random-effects models. Publication bias was assessed using funnel plots and Egger’s test (26). If publication bias was detected, the trim-and-fill method was applied to adjust effect estimates for missing studies (27).

## 5 Results

### 5.1 Study selection

**Figure 1:**
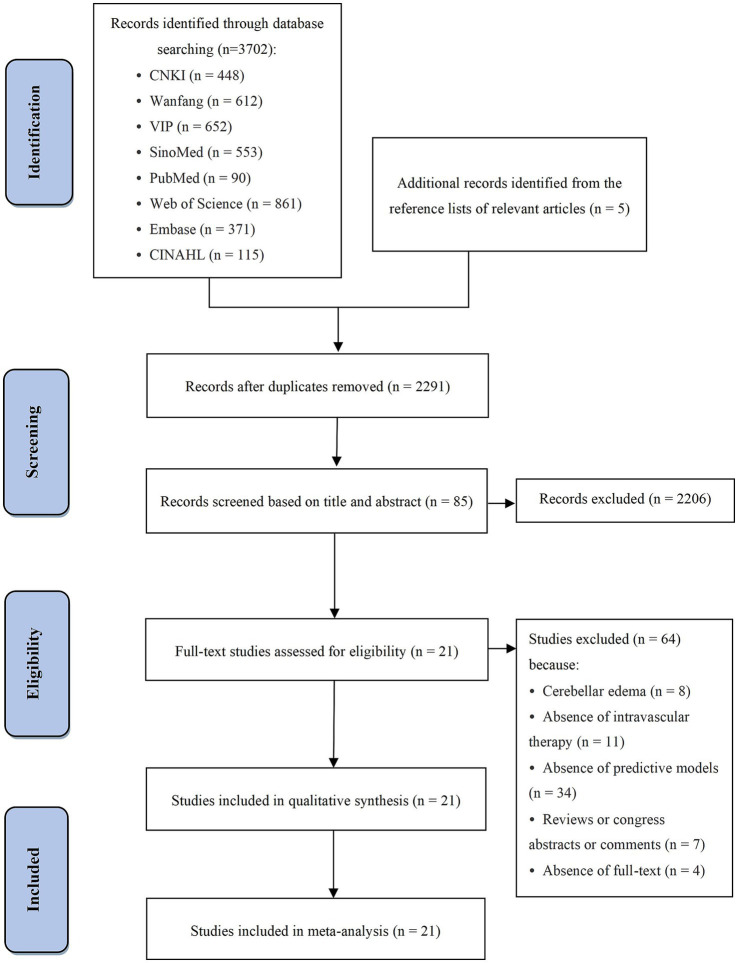
PRISMA 2020 flow diagram of study selection.

A total of 3,702 studies were identified through database searches, with four additional studies retrieved from reference lists. After removing 1,406 duplicates using NoteExpress (version 4.0), 2,291 studies remained. Title and abstract screening excluded 2,206 studies, leaving 85 for full-text review, of which 64 were excluded. Finally, 21 studies (28–48) were included in the analysis. Figure 1 presents the PRISMA 2020 flow diagram.

### 5.2 Study characteristics

Supplementary Table S1 presents a summary of the characteristics of the 21 studies (28–48), published between 2021 and 2024. The sample sizes ranged from 62 to 1,445 patients, and the MCE incidence rate after EVT in acute ACLVOS patients ranged between 5.67 and 50%. Among the 21 studies included, only 6 reported descriptive analyses of patient mortality, with none investigating the specific causes of death in detail. Due to heterogeneity in follow-up durations across studies, a pooled analysis of mortality data was not performed. Mortality was significantly higher in the MCE group, ranging from 39.4 to 64.9%, compared to only 4.1 to 12.7% in the non-MCE control group. In the MCE group, atherosclerotic stroke accounted for 4.05 to 48.15% of cases, while cardioembolic stroke comprised 45.68 to 65.28%. Conversely, in the non-MCE group, the proportions were 7.42 to 50.00% for atherosclerotic stroke and 39.19 to 57.14% for cardioembolic stroke.

Seventeen studies were conducted in China (including five in Chinese and one Chinese dissertation), while two were from the United States and two from the Netherlands. Nine studies focused solely on model development, whereas 12 focused on both development and validation. The majority (13) were case-control studies, with the remaining eight being cohort studies. Seventeen studies targeted acute ACLVOS patients, while four included those with successful recanalization. Regarding treatment, 14 and 7 studies focused on MT and EVT, respectively. Sixteen studies predicted overall MCE probability, while five specifically addressed MCE due to malignant middle cerebral artery infarction (mMCAi). Time frames for MCE prediction varied: five studies assessed within 72 h post-treatment, four assessed within 7 days, five assessed within 5 days, and seven did not specify a timeframe. Diagnostic criteria for MCE varied, with 21 studies employing at least one of 11 different diagnostic criteria.

### 5.3 Model characteristics

Supplementary Table S2 details the characteristics of the 30 MCE prediction models. Among the 12 studies that validated models, three conducted both internal and external validation, three performed only internal validation, and six used external validation. Traditional statistical approaches—logistic regression, least absolute shrinkage and selection operator (LASSO), and 10-fold cross-validation—were employed in 17 studies, while four studies used machine learning techniques such as extreme gradient boosting, random forest, and logistic regression classifiers. Model presentation formats varied: 10 studies used nomograms, two used logistic regression formulas or scales, and nine did not present a model format. Calibration performance was assessed in 14 studies using the Hosmer–Lemeshow test or calibration diagrams, all indicating good fit. AUC values ranged from 0.750 to 0.999. The detailed AUC/C-index values and 95% confidence intervals for the 30 models are available in Supplementary material B.

**Figure 2:**
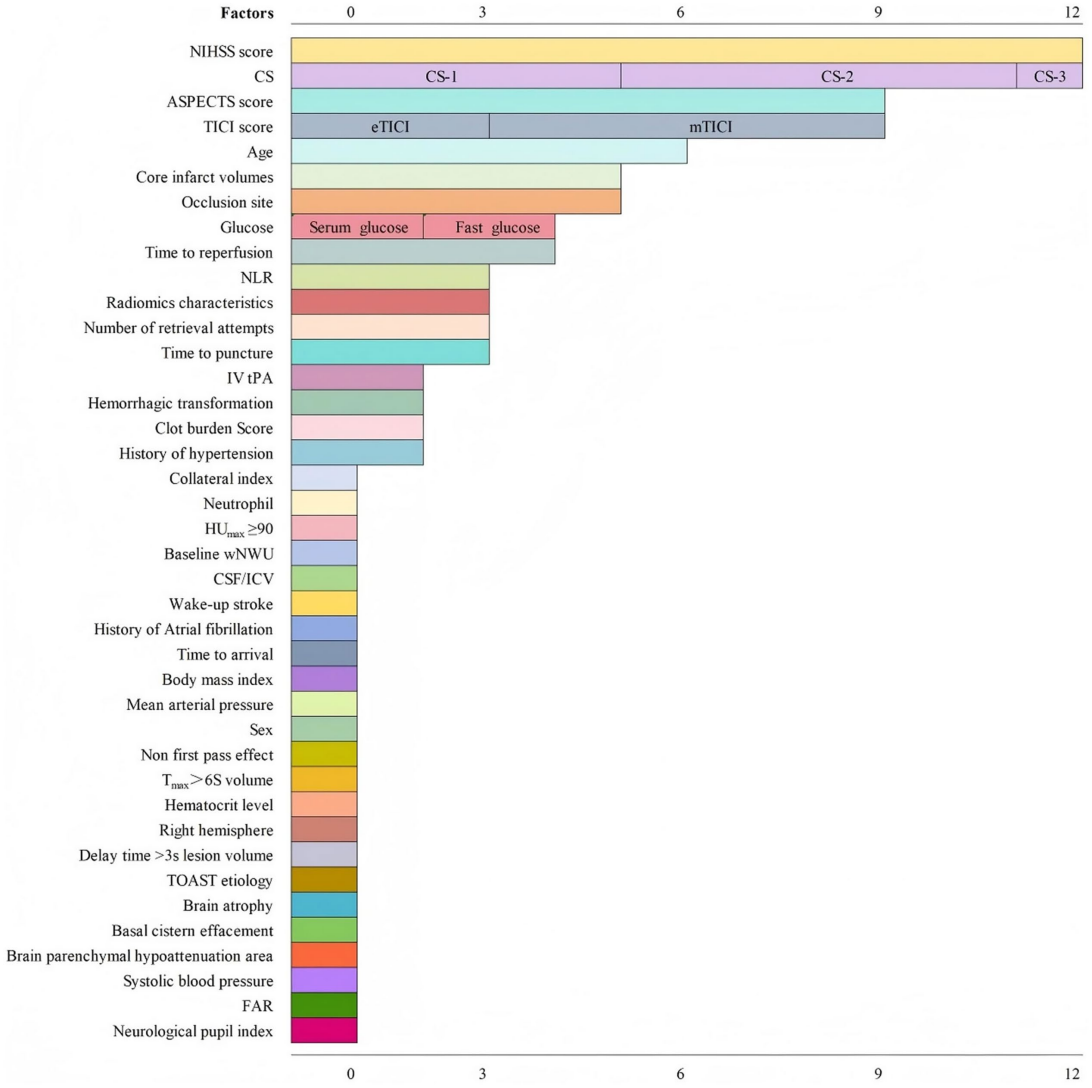
Summary of risk factors of included prediction models.

Figure 2 presents a summary the risk factors identified in the included prediction models, encompassing a total of 40 risk predictors. The most common predictors for MCE were the National Institutes of Health Stroke Scale (NIHSS) score, collateral score (CS), Alberta Stroke Program Early Computed Tomography Score (ASPECTS), Thrombolysis in Cerebral Infarction (TICI) score, and patient age.

### 5.4 Results of quality assessment

Supplementary Table S3 shows the results of quality assessment. Among the 21 included studies, all were identified as having a high risk of bias, and 16 exhibited a high risk of applicability. Detailed quality assessment results are provided in Supplementary material C.

The risk of bias assessment covered four domains: Participants: Eight studies used case–control data sources, introducing a high risk of bias. Predictors: Fourteen studies employed a retrospective design, leading to a high risk of bias. Four studies did not report their research design, preventing bias assessment in this domain. Outcome: Twelve studies were classified as having a high risk of bias, and one had an unclear risk. Nine studies did not report methods to minimize bias in radiological measurement interpretation. Eleven studies failed to apply blinded assessment between predictors and outcomes, and one study did not clarify whether blinding was used. Additionally, two studies did not justify the appropriateness of the time interval between postoperative predictors and outcomes. Analysis: All studies had a high risk of bias. Eighteen studies had insufficient sample sizes, with events per variable (EVP) below 20 in model development or fewer than 100 MCE cases in model validation. Three studies converted continuous variables into categorical variables for analysis. Sixteen studies mishandled missing data: four did not report their approach, while 12 directly excluded missing data. Twenty studies selected predictive factors using univariate analysis. Eight studies did not specify whether they accounted for data complexity, particularly concerning deceased patients. Six studies inadequately assessed model calibration and discrimination, and one study lacked sufficient data for quality assessment. Thirteen studies failed to implement internal validation through self-sampling or cross-validation. Nineteen studies did not provide regression coefficients for predictive factors, limiting the ability to verify consistency with multivariate analysis results.

The risk of applicability assessment included three domains: Participants: Four studies focused exclusively on patients with ACLVOS and successful recanalization. Predictors: One study required specialized measuring instruments for predictor collection, while six relied on advanced imaging analysis software. Outcome: Six studies examined MCE probability specifically related to malignant middle cerebral artery infarction (mMCAi).

### 5.5 Meta-analysis and subgroup analysis of validation models included in the review

Supplementary Table S4 presents the results of the meta-analysis and subgroup analysis of the AUC or C-index. Due to the high heterogeneity across studies (*I*^2^ > 50%), a random-effects model was used for both analyses. Subgroup analysis was performed based on participant characteristics, prediction outcomes, prediction time range, and model development or validation methods. A meta-analysis was not conducted for internal validation data due to insufficient reporting. Corresponding forest plots for the meta-analysis and subgroup analysis are available in Supplementary material D.

**Figure 3:**
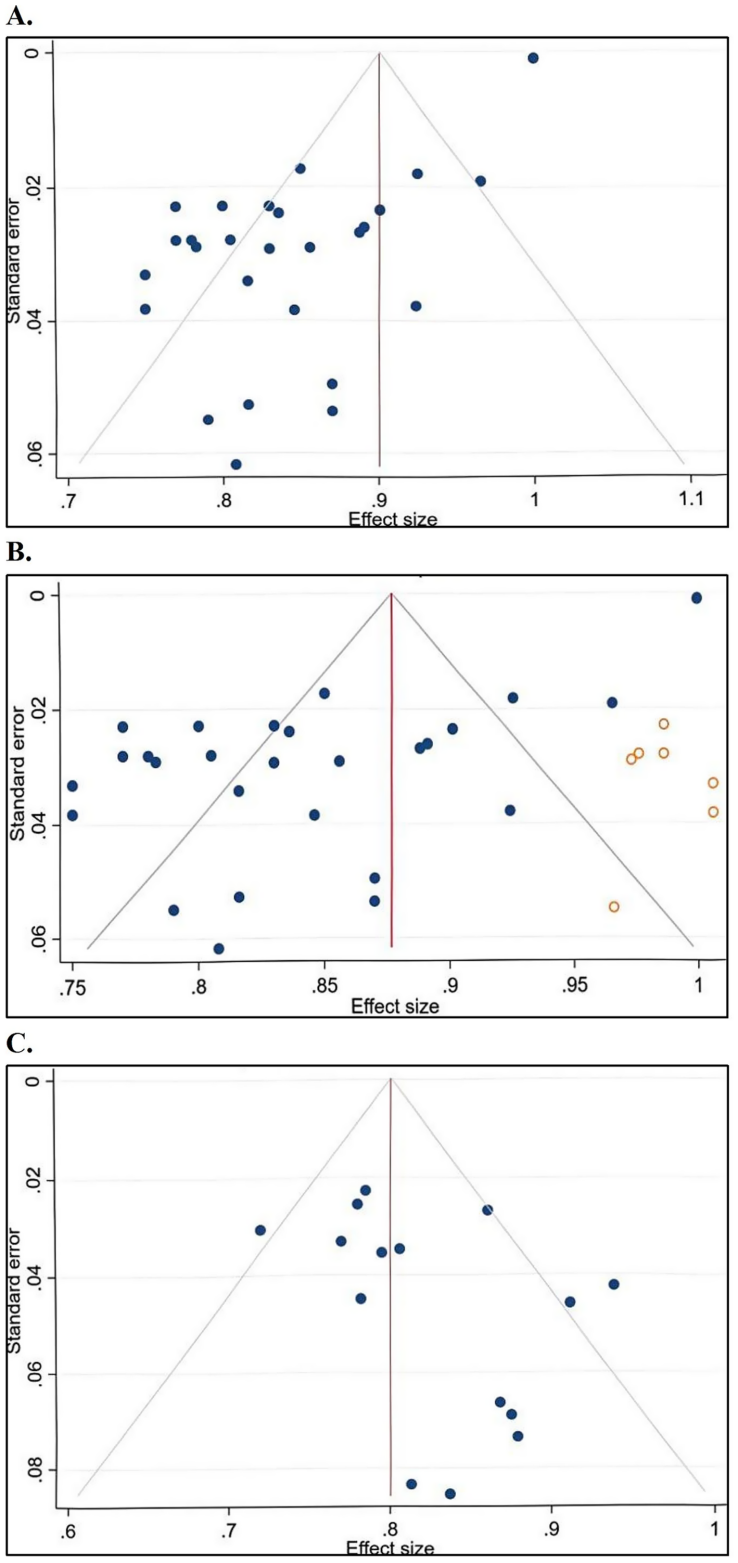
**A**: funnel plot of model development data; **B**: funnel plot including imputed data points of model development data; **C**: funnel plot of model external validation data.

For model development data, 28 models from 19 studies were included, while studies conducted by Frans Kauw and Jun Tong were excluded due to insufficient data. The meta-analyzed AUC/C index was 0.86 (95% confidence interval [CI]: 0.85–0.88). Egger’s test (*t* = −11.9, *p* < 0.001), and the funnel plot indicated publication bias. After trimming and filling analysis, the corrected AUC/C index slightly increased (effect size = 0.88, 95% CI: 0.85–0.90). The funnel plot for model development data is shown in Figure 3A, and the funnel plot incorporating imputed data is shown in Figure 3B.

For external validation data, only 16 models from 9 studies were included. The meta-analyzed AUC/C index was 0.81 (95% CI: 0.78–0.84). Egger’s test (*t* = 1.78, *p* = 0.096) and the funnel plot indicated no publication bias. The funnel plot for external validation data is shown in Figure 3C.

## 6 Discussion

In this systematic review, we identified 30 MCE prediction models across 21 studies focusing on EVT ACLVOS patients. Despite demonstrating good predictive performance, all included studies exhibited a high risk of bias. Given the heterogeneity among models, we conducted subgroup analyses. Using data from internal and external validations, a meta-analysis was performed based on participant characteristics, prediction outcomes, study timeframes, and external validation methodologies. Notably, significant variability exists in MCE outcome definitions. Most studies defined MCE based on imaging evidence, clinical features, decompressive hemicraniectomy necessity, mortality, consciousness deterioration, and NIHSS score increases.

For model development studies, significant publication bias was detected, suggesting that studies with poorer performance are under-represented. This is a common issue in predictive modeling research where negative results are less frequently published. The trim-and-fill adjusted AUC showed only a marginal increase, indicating that the overall excellent performance is likely robust. In contrast, external validation data exhibited no significant publication bias. This discrepancy is expected, as validation studies inherently test generalizability, often leading to a predictable performance attenuation. The absence of bias in validation literature is reassuring, suggesting that evidence on model transportability is relatively complete. Collectively, while existing models show promise, their development-phase results should be interpreted with caution due to publication bias. Future work should prioritize prospective study registration and the publication of all validation outcomes to mitigate this issue.

The significantly higher mortality in the MCE group (39.4–64.9%) corresponds to the known severe outcomes of malignant edema, such as intracranial hypertension and cerebral herniation. This mortality pattern may be explained by the etiological distribution: MCE patients showed a higher proportion of cardioembolic stroke (45.68–65.28%) compared to non-MCE patients (39.19–57.14%). These findings align with existing evidence that cardioembolic and atherothrombotic strokes carry the greatest risk of malignant edema and the highest in-hospital mortality. Cardioembolic strokes, in particular, are linked to larger infarct volumes and more severe edema, resulting in poorer short-term prognosis, as reported in prior studies (5). Thus, the overrepresentation of this high-risk subtype substantially contributes to the devastating mortality associated with MCE.

Among the 40 identified risk predictors, the five most frequently reported variables were the NIHSS score, collateral status (CS), ASPECTS score, TICI score, and age. Clinicians should remain vigilant regarding these factors. NIHSS score, a widely used neurological function and prognosis assessment tool in stroke patients (49, 50), is particularly relevant, as MCE is characterized by progressive neurological deterioration (30). A high NIHSS score suggests extensive cerebral infarction and an elevated MCE risk (40). Collateral status (CS) is a key measure of collateral circulation quality in stroke patients (51). This review examined three CS grading standards—Tan et al.’s (52), ASITN/SIR (53), and Christoforidis (54). Adequate collateral circulation prolongs blood supply to ischemic regions, preserving viable brain tissue and reducing MCE risk (28). The ASPECTS score is used to assess ischemic changes in 10 cerebral artery supply regions (55). A low ASPECTS score correlates with a higher MCE risk. Automated software applications may enhance the objectivity of ASPECTS scoring (30). The TICI score is used to evaluate vascular recanalization in ischemic stroke patients (53). Both extended Thrombolysis in Cerebral Infarction (eTICI) (56) and modified Thrombolysis in Cerebral Infarction (mTICI) (57) have been used for MCE prediction. Successful recanalization not only reduces MCE risk but also enhances EVT clinical benefits (48). Interestingly, advanced age has been suggested as a protective factor against MCE (48), as age-related cerebral atrophy may provide additional buffering capacity against brain swelling (46). However, the optimal age cutoff for prediction remains unclear, and this view is not without contradiction. In fact, some studies indicate that older patients may experience more severe infarctions, thereby predisposing them to MCE (58, 59). Given the distinct demographics and risk profiles of the very old stroke population, further research is warranted to clarify the complex relationship between advanced age and MCE risk (60). Previous study suggested that female sex may be a risk factor for increased stroke severity and worse prognosis (61). However, among the prediction models included in the study, only one incorporated female sex as a predictor variable. The pathophysiological mechanisms and regulatory pathways linking sex to the development of MCE still require further investigation to be fully elucidated. Given the alignment of its predictors with the most frequent and clinically accessible variables identified in our review, the models developed by Huang et al. (40) and Guo et al. (42) appear to be the most immediately applicable. Its adoption should be coupled with prospective, large-sample external validation and continuous parameter optimization.

The current model requires methodological enhancements, particularly in mitigating bias. To improve reliability, avoid using multivariate analysis results for model development. Most studies selected statistically significant univariate predictors, potentially overlooking crucial variables. An exception is the study conducted by Hoffman et al. (33), which applied variance thresholding and recursive feature elimination for predictor selection. Ensure full transparency by reporting all predictive factors and regression coefficients. The omission of regression coefficients in nearly all studies hinders consistency verification with multivariate analysis results. An adequate sample size should be used, maintaining a minimum ratio of 20 outcome events per candidate predictor, to prevent overfitting. Missing data should be handled using multiple imputation rather than direct deletion to minimize bias and enhance model robustness. Data from cohort studies, randomized controlled trials, or nested case–control studies should be prioritized for model development. Strengthening research design will reduce bias and confounding effects, thereby improving model stability and reliability.

## 7 Limitations

This review had several limitations. The broad search strategy required extensive study filtering. Extracting multiple models from individual studies may have introduced variability, but this was necessary to prevent overestimation from analyzing only top-performing models. Most of the included studies were published in Chinese, potentially limiting model applicability. Additionally, restricting inclusion to studies published in Chinese and English may have introduced language bias.

## 8 Implications and recommendations for practice and future research

Based on this study, a standardized definition of MCE should be developed in the future. There are few predictive models developed for nursing, and many models use imaging indicators, which may limit nurses’ use of predictive models. Based on the results of this study, more nursing related indicators can be included to develop a nursing prediction model for MCE. Moreover, future research should extend beyond risk prediction toward implementing precision management strategies. This involves utilizing individual risk stratification to identify patients most likely to benefit from specific interventions, such as neuroprotective agents, decompressive craniectomy, or dehydration therapies (62). A deeper understanding of the high-risk factors and underlying pathophysiological mechanisms of malignant cerebral edema is also needed, as these insights are critical for discovering novel therapeutic targets. Finally, before any prediction model can be widely adopted, it must undergo rigorous validation in large-scale, independent, prospective cohorts to confirm its generalizability and clinical utility.

## 9 Conclusion

We identified 40 risk predictors and 30 MCE prediction models from 21 studies. Predicting MCE risk after EVT in acute ACLVOS patients is feasible. However, all included studies had a high risk of bias, and only five exhibited high applicability per PROBAST criteria. Future research should emphasize rigorous study design, larger sample sizes, comprehensive data reporting, and multicenter external validation to enhance model performance.

## Data Availability

The datasets presented in this study can be found in online repositories. The names of the repository/repositories and accession number(s) can be found in the article/Supplementary material.
