# Supplementary material for: Risk prediction models for malignant cerebral edema after endovascular therapy in patients with acute anterior circulation large vessel occlusion stroke: a systematic review and meta-analysis
Source: Front Neurol. 2026 Feb 5;17:1686413. doi: 10.3389/fneur.2026.1686413 (PMC12916362; doi:10.3389/fneur.2026.1686413)
Supplement: Supplementary file 1 [file Supplementary_file_1.docx]

**Supplementary material A. Detailed search strategies and specific queries for each database**

| **Database** | **#** | **Search Query** | **Results** |
| --- | --- | --- | --- |
| **PubMed** | 1 | ((((malignant cerebral edema[Title/Abstract]) OR (severe cerebral edema[Title/Abstract])) OR (malignant edema[Title/Abstract])) OR (cerebral edema[Title/Abstract])) OR (life-threatening edema[Title/Abstract]) | 7,062 |
|  | 2 | (((((((((((neurological critical illness[Title/Abstract]) OR (stroke[Title/Abstract])) OR (cerebral infarction[Title/Abstract])) OR (cerebral ischemia[Title/Abstract])) OR (cerebral blood vessels[Title/Abstract])) OR (intracranial arteries[Title/Abstract])) OR (intracranial veins[Title/Abstract])) OR (cerebral arteries[Title/Abstract])) OR (cerebral veins[Title/Abstract])) OR (stenosis[Title/Abstract])) OR (malformations[Title/Abstract])) OR (occlusion[Title/Abstract]) | 744,588 |
|  | 3 | (((((endovascular therapy[Title/Abstract]) OR (thrombolysis[Title/Abstract])) OR (thrombectomy[Title/Abstract])) OR (intervention[Title/Abstract])) OR (bridging[Title/Abstract])) OR (stenting[Title/Abstract]) | 995,887 |
|  | 4 | (((((((((((((((((((((prediction[Title/Abstract]) OR (early warning[Title/Abstract])) OR (impact[Title/Abstract])) OR (factors[Title/Abstract])) OR (risks[Title/Abstract])) OR (models[Title/Abstract])) OR (tools[Title/Abstract])) OR (column[Title/Abstract])) OR (norman[Title/Abstract])) OR (stratification[Title/Abstract])) OR (validation[Title/Abstract])) OR (screening[Title/Abstract])) OR (machine learning[Title/Abstract])) OR (rules[Title/Abstract])) OR (scoring[Title/Abstract])) OR (ROC curves[Title/Abstract])) OR (deep learning[Title/Abstract])) OR (decision support[Title/Abstract])) OR (support quantitative machines[Title/Abstract])) OR (neural networks[Title/Abstract])) OR (artificial intelligence[Title/Abstract])) OR (random forests[Title/Abstract]) | 7,034,757 |
|  | 5 | #1 AND #2 | 1,703 |
|  | 6 | #3 AND #5 | 246 |
|  | 7 | #4 AND #6 | 90 |
| **Web of Science** | 1 | TS=(malignant cerebral edema OR severe cerebral edema OR malignant edema OR cerebral edema OR life-threatening edema) | 18,750 |
|  | 2 | TS=(neurological critical illness OR stroke OR cerebral infarction OR cerebral ischemia OR cerebral blood vessels OR intracranial arteries OR intracranial veins OR cerebral arteries OR cerebral veins OR stenosis OR malformations OR occlusion) | 853,665 |
|  | 3 | TS=(endovascular therapy OR thrombolysis OR thrombectomy OR intervention OR bridging OR stenting) | 1,644,094 |
|  | 4 | TS=(prediction OR early warning OR impact OR factors OR risks OR models OR tools OR column OR norman OR stratification OR validation OR screening OR machine learning OR rules OR scoring OR ROC curves OR deep learning OR decision support OR support quantitative machines OR neural networks OR artificial intelligence OR random forests) | 19,408,445 |
|  | 5 | #1 AND #2 | 8,799 |
|  | 6 | #3 AND #5 | 1,195 |
|  | 7 | #4 AND #6 | 861 |
| **Database** | **#** | **Search Query** | **Results** |
| **Embase** | 1 | (malignant cerebral edema OR severe cerebral edema OR malignant edema OR cerebral edema OR life-threatening edema):ab,ti | 25,525 |
|  | 2 | (neurological critical illness OR stroke OR cerebral infarction OR cerebral ischemia OR cerebral blood vessels OR intracranial arteries OR intracranial veins OR cerebral arteries OR cerebral veins OR stenosis OR malformations OR occlusion):ab,ti | 1,109,601 |
|  | 3 | (endovascular therapy OR thrombolysis OR thrombectomy OR intervention OR bridging OR stenting):ab,ti | 1,388,059 |
|  | 4 | (prediction OR early warning OR impact OR factors OR risks OR models OR tools OR column OR norman OR stratification OR validation OR screening OR machine learning OR rules OR scoring OR ROC curves OR deep learning OR decision support OR support quantitative machines OR neural networks OR artificial intelligence OR random forests):ab,ti | 9,331,005 |
|  | 5 | #1 AND #2 | 9,469 |
|  | 6 | #3 AND #5 | 1,124 |
|  | 7 | #4 AND #6 | 371 |
| **CINAHL** | 1 | (malignant cerebral edema OR severe cerebral edema OR malignant edema OR cerebral edema OR life-threatening edema) | 3,124 |
|  | 2 | (neurological critical illness OR stroke OR cerebral infarction OR cerebral ischemia OR cerebral blood vessels OR intracranial arteries OR intracranial veins OR cerebral arteries OR cerebral veins OR stenosis OR malformations OR occlusion) | 235,040 |
|  | 3 | (endovascular therapy OR thrombolysis OR thrombectomy OR intervention OR bridging OR stenting) | 617,285 |
|  | 4 | (prediction OR early warning OR impact OR factors OR risks OR models OR tools OR column OR norman OR stratification OR validation OR screening OR machine learning OR rules OR scoring OR ROC curves OR deep learning OR decision support OR support quantitative machines OR neural networks OR artificial intelligence OR random forests) | 3,462,769 |
|  | 5 | #1 AND #2 | 608 |
|  | 6 | #3 AND #5 | 115 |
|  | 7 | #4 AND #6 | 115 |
| **CNKI** | 1 | 恶性脑水肿 + 重度脑水肿 + 严重脑水肿 + 脑水肿 | 3805 |
|  | 2 | 神经重症 OR 卒中 OR 脑梗死 OR 脑缺血 OR 脑出血 OR 蛛网膜下腔出血 OR 动脉瘤 OR 脑血管 OR 脑小血管病 OR 颅内动脉 OR 颅内静脉 OR 脑动脉 OR 脑静脉 OR 动脉狭窄 OR 动脉畸形 OR 闭塞 OR 血肿 OR 溶栓 OR 取栓 OR 介入 OR 桥接 OR 转孔 OR 支架植入 OR 造影 OR 颈动脉夹层 OR 血管内治疗 | 611,832 |
|  | 3 | 预测 OR 预警 OR 影响 OR 因素 OR 因子 OR 风险 OR 模型 OR 工具 OR 列线图 OR 诺曼图 OR 分层 OR 验证 OR 筛查OR 机器学习 OR 规则 OR 评分 OR 识别 OR ROC曲线 OR 深度学习 OR 决策支持 OR 支持定量机 OR 神经网络 OR 人工智能 OR 随机森林 | 6,606,484 |
|  | 4 | #1 AND #2 AND #3 | 448 |
| **Database** | **#** | **Search Query** | **Results** |
| **Wanfang** | #1 | 恶性脑水肿 OR 重度脑水肿 OR 严重脑水肿 OR 脑水肿 | 4,248 |
|  | #2 | 神经重症 OR 卒中 OR 脑梗死 OR 脑缺血 OR 脑出血 OR 蛛网膜下腔出血 OR 动脉瘤 OR 脑血管 OR 脑小血管病 OR 颅内动脉 OR 颅内静脉 OR 脑动脉 OR 脑静脉 OR 动脉狭窄 OR 动脉畸形 OR 闭塞 OR 血肿 OR 溶栓 OR 取栓 OR 介入 OR 桥接 OR 转孔 OR 支架植入 OR 造影 OR 颈动脉夹层 OR 血管内治疗 | 707,196 |
|  | #3 | 预测 OR 预警 OR 影响 OR 因素 OR 因子 OR 风险 OR 模型 OR 工具 OR 列线图 OR 诺曼图 OR 分层 OR 验证 OR 筛查OR 机器学习 OR 规则 OR 评分 OR 识别 OR ROC曲线 OR 深度学习 OR 决策支持 OR 支持定量机 OR 神经网络 OR 人工智能 OR 随机森林 | 6,855,553 |
|  | #4 | #1 AND #2 AND #3 | 612 |
| **VIP** | #1 | 恶性脑水肿 OR 重度脑水肿 OR 严重脑水肿 OR 脑水肿 | 4,363 |
|  | #2 | 神经重症 OR 卒中 OR 脑梗死 OR 脑缺血 OR 脑出血 OR 蛛网膜下腔出血 OR 动脉瘤 OR 脑血管 OR 脑小血管病 OR 颅内动脉 OR 颅内静脉 OR 脑动脉 OR 脑静脉 OR 动脉狭窄 OR 动脉畸形 OR 闭塞 OR 血肿 OR 溶栓 OR 取栓 OR 介入 OR 桥接 OR 转孔 OR 支架植入 OR 造影 OR 颈动脉夹层 OR 血管内治疗 | 609,683 |
|  | #3 | 预测 OR 预警 OR 影响 OR 因素 OR 因子 OR 风险 OR 模型 OR 工具 OR 列线图 OR 诺曼图 OR 分层 OR 验证 OR 筛查OR 机器学习 OR 规则 OR 评分 OR 识别 OR ROC曲线 OR 深度学习 OR 决策支持 OR 支持定量机 OR 神经网络 OR 人工智能 OR 随机森林 | 6,602,003 |
|  | #4 | #1 AND #2 AND #3 | 652 |
| **SinoMed** | #1 | 恶性脑水肿 OR 重度脑水肿 OR 严重脑水肿 OR 脑水肿 | 3901 |
|  | #2 | 神经重症 OR 卒中 OR 脑梗死 OR 脑缺血 OR 脑出血 OR 蛛网膜下腔出血 OR 动脉瘤 OR 脑血管 OR 脑小血管病 OR 颅内动脉 OR 颅内静脉 OR 脑动脉 OR 脑静脉 OR 动脉狭窄 OR 动脉畸形 OR 闭塞 OR 血肿 OR 溶栓 OR 取栓 OR 介入 OR 桥接 OR 转孔 OR 支架植入 OR 造影 OR 颈动脉夹层 OR 血管内治疗 | 528,758 |
|  | #3 | 预测 OR 预警 OR 影响 OR 因素 OR 因子 OR 风险 OR 模型 OR 工具 OR 列线图 OR 诺曼图 OR 分层 OR 验证 OR 筛查OR 机器学习 OR 规则 OR 评分 OR 识别 OR ROC曲线 OR 深度学习 OR 决策支持 OR 支持定量机 OR 神经网络 OR 人工智能 OR 随机森林 | 1,701,307 |
|  | #4 | #1 AND #2 AND #3 | 553 |
