# Supplementary material for: Risk prediction models for malignant cerebral edema after endovascular therapy in patients with acute anterior circulation large vessel occlusion stroke: a systematic review and meta-analysis
Source: Front Neurol. 2026 Feb 5;17:1686413. doi: 10.3389/fneur.2026.1686413 (PMC12916362; doi:10.3389/fneur.2026.1686413)
Supplement: Supplementary file 2 [file Supplementary_file_2.docx]

**Supplementary material B. Area under the curve (AUC) / C index of included models.**

| Author / Year | Model development method | Name of prediction model | Model Development | | | Internal Validation | | | External Validation | | |
| --- | --- | --- | --- | --- | --- | --- | --- | --- | --- | --- | --- |
|  |  |  | AUC | 95%CI- | 95%CI+ | AUC | 95%CI- | 95%CI+ | AUC | 95%CI- | 95%CI+ |
| Huiyuan Wang / 2024 | logistic regression | nomogram | 0.891 | 0.840 | 0.942 | 0.849 | 0.726 | 0.973 | 0.911 | 0.819 | 0.999 |
| Sheng Hu / 2024 | XGBoost | clinical model | 0.790 | 0.683 | 0.898 | NR | NR | NR | 0.868 | 0.738 | 0.998 |
| Sheng Hu / 2024 | XGBoost | radiomics model | 0.999 | 0.996 | 1.000 | NR | NR | NR | 0.875 | 0.730 | 1.000 |
| Sheng Hu / 2024 | XGBoost | combined nomograms | 0.999 | 0.996 | 1.000 | NR | NR | NR | 0.938 | 0.834 | 1.000 |
| Haoli Xu / 2024 | logistic regression | combined model | 0.800 | 0.750 | 0.840 | NR | NR | NR | NR | NR | NR |
| Haoli Xu / 2024 | logistic regression | basic model | 0.770 | 0.720 | 0.810 | NR | NR | NR | NR | NR | NR |
| Xiaoquan Xu / 2023 | logistic regression | combined predictive model | 0.836 | 0.789 | 0.883 | NR | NR | NR | NR | NR | NR |
| Frans Kauw / 2023 | logistic regression | extended model | 0.876 | NR | NR | NR | NR | NR | NR | NR | NR |
| Haydn Hoffman / 2023 | machine learning | random forest | 0.750 | 0.680 | 0.810 | 0.840 | NR | NR | 0.780 | 0.730 | 0.830 |
| Haydn Hoffman / 2023 | machine learning | neural network | 0.780 | 0.720 | 0.830 | 0.740 | NR | NR | 0.780 | 0.730 | 0.830 |
| Haydn Hoffman / 2023 | machine learning | support vector machine | 0.770 | 0.720 | 0.830 | 0.840 | NR | NR | 0.770 | 0.700 | 0.830 |
| Haydn Hoffman / 2023 | machine learning | logistic regression | 0.750 | 0.670 | 0.820 | NR | NR | NR | 0.720 | 0.660 | 0.780 |
| Liyong Zhang / 2023 | logistic regression | nomogram | 0.846 | 0.770 | 0.921 | NR | NR | NR | NR | NR | NR |
| Jun Tong / 2023 | logistic regression | nomogram | 0.959 | NR | NR | NR | NR | NR | 0.889 | NR | NR |
| Yuxuan He / 2023 | logistic regression | nomogram | 0.901 | 0.848 | 0.940 | NR | NR | NR | NR | NR | NR |
| Xi Li / 2023 | logistic regression | nomogram | 0.830 | 0.773 | 0.888 | NR | NR | NR | 0.860 | 0.808 | 0.913 |
| Xuehua Wen / 2023 | logistic regression | the combined model | 0.924 | 0.850 | 0.998 | NR | NR | NR | 0.879 | 0.712 | 1.000 |
| Xuehua Wen / 2023 | LASSO | the radiomics signature | 0.870 | 0.773 | 0.967 | NR | NR | NR | 0.837 | 0.665 | 1.000 |
| Xuehua Wen / 2023 | logistic regression | routine visual radiological model | 0.808 | 0.687 | 0.929 | NR | NR | NR | 0.813 | 0.649 | 0.976 |
| Huigui Zhao / 2023 | logistic regression | risk prediction scoring model | 0.888 | 0.836 | 0.941 | NR | NR | NR | NR | NR | NR |
| Xianjun Huang / 2022 | logistic regression | ACORNS grading scale | 0.850 | 0.816 | 0.884 | 0.874 | 0.821 | 0.926 | 0.785 | 0.740 | 0.829 |

| Author / Year | Model development method | Name of prediction models | Model Development | | | Internal Validation | | | External Validation | | |
| --- | --- | --- | --- | --- | --- | --- | --- | --- | --- | --- | --- |
|  |  |  | AUC | 95%CI- | 95%CI+ | AUC | 95%CI- | 95%CI+ | AUC | 95%CI- | 95%CI+ |
| Qianmei Jiang / 2022 | logistic regression | New nomogram | 0.925 | 0.890 | 0.961 | 0.915 | NR | NR | NR | NR | NR |
| Wenting Guo / 2022 | logistic regression | nomogram | 0.783 | 0.726 | 0.840 | NR | NR | NR | 0.806 | 0.738 | 0.874 |
| Ning Li / 2022 | logistic regression | nomogram | 0.965 | 0.915 | 0.990 | 0.965 | NR | NR | NR | NR | NR |
| Jun Cheng / 2022 | logistic regression | logistic regression | 0.816 | 0.749 | 0.883 | NR | NR | NR | 0.782 | 0.709 | 0.885 |
| Jun Cheng / 2022 | XGBoost | XGBoost | 0.856 | 0.799 | 0.913 | NR | NR | NR | 0.795 | 0.725 | 0.864 |
| Liangxu Xiang / 2022 | logistic regression | combined model | 0.816 | 0.697 | 0.903 | NR | NR | NR | NR | NR | NR |
| Marie Louise E Bernsen / 2021 | logistic regression | basic model | 0.830 | 0.790 | 0.880 | NR | NR | NR | NR | NR | NR |
| Ehsan Dowlati / 2021 | logistic regression | multivariate regression model | 0.870 | 0.735 | 0.945 | NR | NR | NR | NR | NR | NR |
| Mingyang Du / 2020 | logistic regression | nomogram | 0.805 | 0.750 | 0.860 | 0.805 | NR | NR | NR | NR | NR |

AUC: area under the curve; CI: confidence interval; XGBoost: extreme gradient boosting; NR: not reported; LASSO: least absolute shrinkage and selection operator;
