# Supplementary material for: Risk prediction models for malignant cerebral edema after endovascular therapy in patients with acute anterior circulation large vessel occlusion stroke: a systematic review and meta-analysis
Source: Front Neurol. 2026 Feb 5;17:1686413. doi: 10.3389/fneur.2026.1686413 (PMC12916362; doi:10.3389/fneur.2026.1686413)
Supplement: Supplementary file 3 [file Supplementary_file_3.docx]

**Supplementary material C. Detailed results of risk of bias assessment.**

| **Study** | **Participants** | | **Predictors** | | | **Outcome** | | | | | | **Analysis** | | | | | | | | |
| --- | --- | --- | --- | --- | --- | --- | --- | --- | --- | --- | --- | --- | --- | --- | --- | --- | --- | --- | --- | --- |
|  | **1.1** | **1.2** | **2.1** | **2.2** | **2.3** | **3.1** | **3.2** | **3.3** | **3.4** | **3.5** | **3.6** | **4.1** | **4.2** | **4.3** | **4.4** | **4.5** | **4.6** | **4.7** | **4.8** | **4.9** |
| Huiyuan Wang / 2024 | Y | Y | Y | N | Y | Y | Y | Y | Y | Y | Y | N | Y | Y | N | N | Y | Y | Y | ? |
| Sheng Hu / 2024 | Y | Y | Y | ? | Y | Y | Y | Y | Y | Y | Y | N | N | Y | N | N | Y | N | Y | ? |
| Haoli Xu / 2024 | N | Y | Y | N | Y | Y | Y | Y | ? | N | Y | N | Y | Y | N | N | Y | Y | N | ? |
| Xiaoquan Xu / 2023 | Y | Y | Y | Y | Y | Y | Y | Y | Y | Y | Y | N | Y | Y | Y | N | ? | Y | N | ? |
| Frans Kauw / 2023 | Y | Y | Y | Y | Y | Y | Y | Y | Y | Y | Y | Y | Y | Y | Y | N | ? | ? | N | ? |
| Haydn Hoffman / 2023 | Y | Y | Y | N | Y | Y | Y | Y | ? | N | Y | N | Y | Y | Y | Y | Y | N | Y | ? |
| Zhang Liyong / 2023 | N | Y | Y | N | Y | Y | Y | Y | ? | N | Y | N | Y | Y | N | N | Y | N | Y | ? |
| Tong Jun / 2023 | N | Y | Y | N | Y | Y | Y | Y | Y | Y | Y | N | Y | Y | N | N | ? | N | N | ? |
| Yuxuan He / 2023 | N | Y | Y | N | Y | Y | Y | Y | Y | N | Y | N | N | Y | N | N | ? | Y | N | ? |
| Li Xi / 2023 | N | Y | Y | ? | Y | Y | Y | Y | ? | N | Y | N | Y | Y | N | N | Y | Y | N | ? |
| Xuehua Wen / 2023 | N | Y | Y | N | Y | Y | Y | Y | Y | N | Y | N | Y | Y | N | N | Y | Y | N | ? |
| Zhao Huigui / 2023 | N | Y | Y | N | Y | Y | Y | Y | ? | N | ? | N | Y | Y | N | N | ? | Y | N | Y |
| Xianjun Huang / 2022 | Y | Y | Y | ? | Y | Y | Y | Y | Y | Y | Y | Y | Y | Y | Y | N | Y | Y | Y | Y |
| Jiang Qianmei / 2022 | N | Y | Y | N | Y | Y | Y | Y | Y | Y | Y | N | Y | Y | N | N | Y | Y | Y | ? |
| Wenting Guo / 2022 | Y | Y | Y | ? | Y | Y | Y | Y | Y | ? | Y | N | N | Y | N | N | Y | Y | N | ? |
| Li Ning / 2022 | N | Y | Y | N | Y | Y | Y | Y | ? | N | ? | N | Y | Y | N | N | Y | Y | Y | ? |
| Cheng Jun / 2022 | N | Y | Y | N | Y | Y | Y | Y | ? | N | Y | N | Y | Y | N | N | Y | Y | N | ? |
| Liangxu Xiang / 2022 | N | Y | Y | N | Y | Y | Y | Y | ? | N | Y | N | Y | Y | N | N | ? | N | N | ? |
| Marie Louise E Bernsen / 2021 | Y | Y | Y | Y | Y | Y | Y | Y | Y | Y | Y | Y | Y | Y | Y | N | Y | Y | N | ? |
| Ehsan Dowlati / 2021 | N | Y | Y | N | Y | Y | Y | Y | ? | N | Y | N | Y | Y | N | N | ? | N | N | ? |
| Mingyang Du / 2020 | N | Y | Y | N | Y | Y | Y | Y | Y | Y | Y | N | Y | Y | N | N | ? | Y | Y | ? |

Y: Yes, indicates low ROB; N: No, indicates high ROB; ?: Unknown, indicates unclear ROB.
