# Supplementary material for: Risk prediction models for malignant cerebral edema after endovascular therapy in patients with acute anterior circulation large vessel occlusion stroke: a systematic review and meta-analysis
Source: Front Neurol. 2026 Feb 5;17:1686413. doi: 10.3389/fneur.2026.1686413 (PMC12916362; doi:10.3389/fneur.2026.1686413)
Supplement: Supplementary file 4 [file Supplementary_file_4.docx]

**Supplementary material D. The AUC meta-analysis and subgroup analysis.**

1. **B.**

**
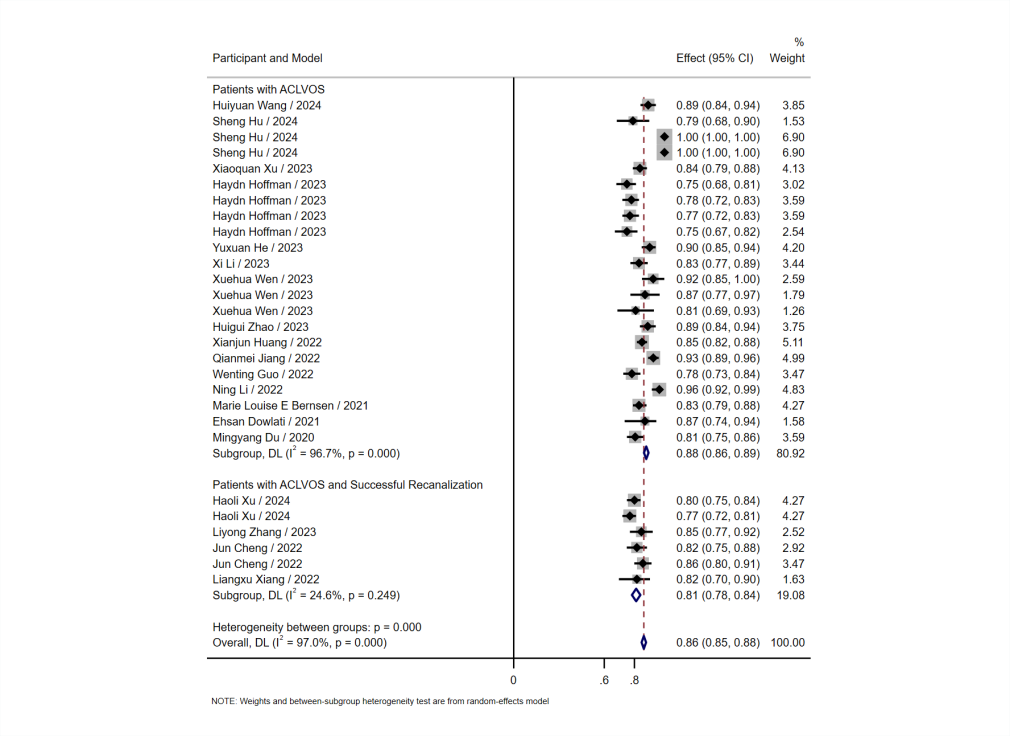
**
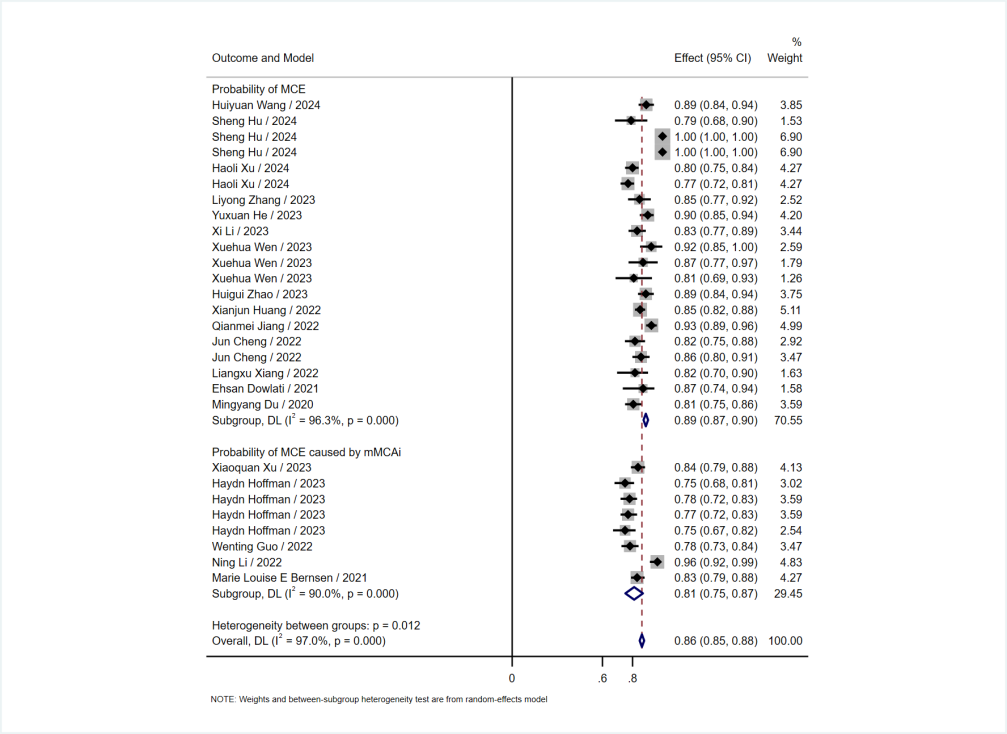


**C. D.**


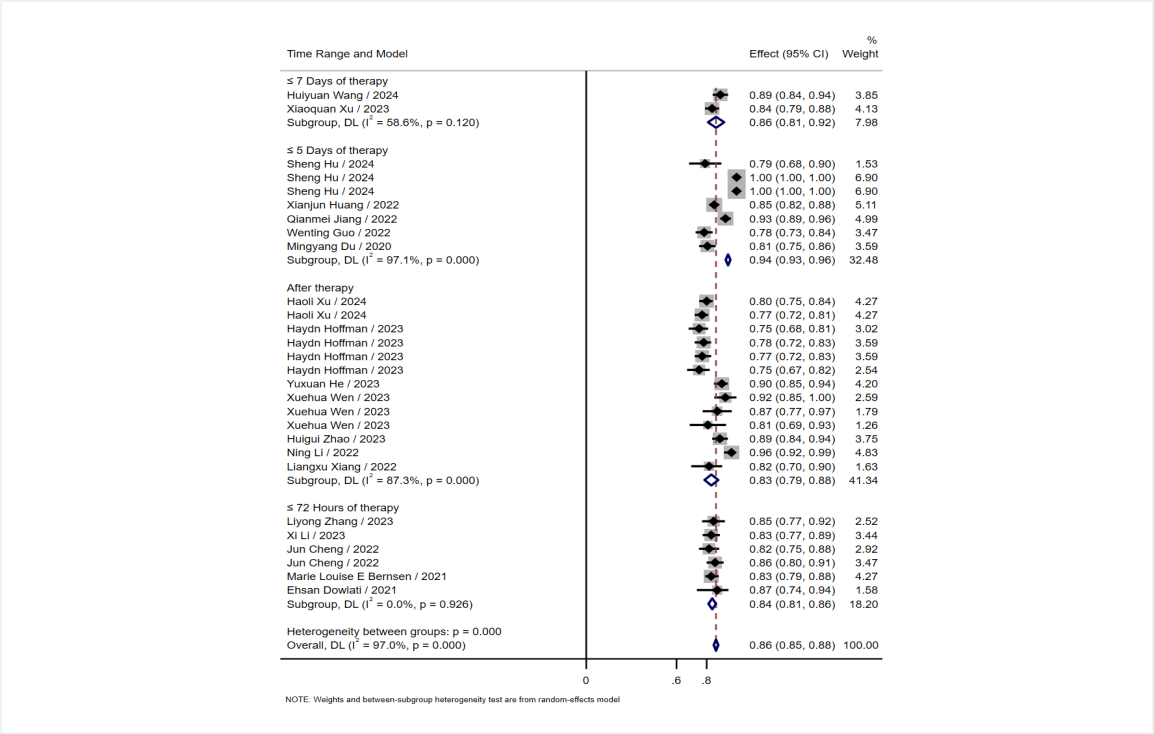

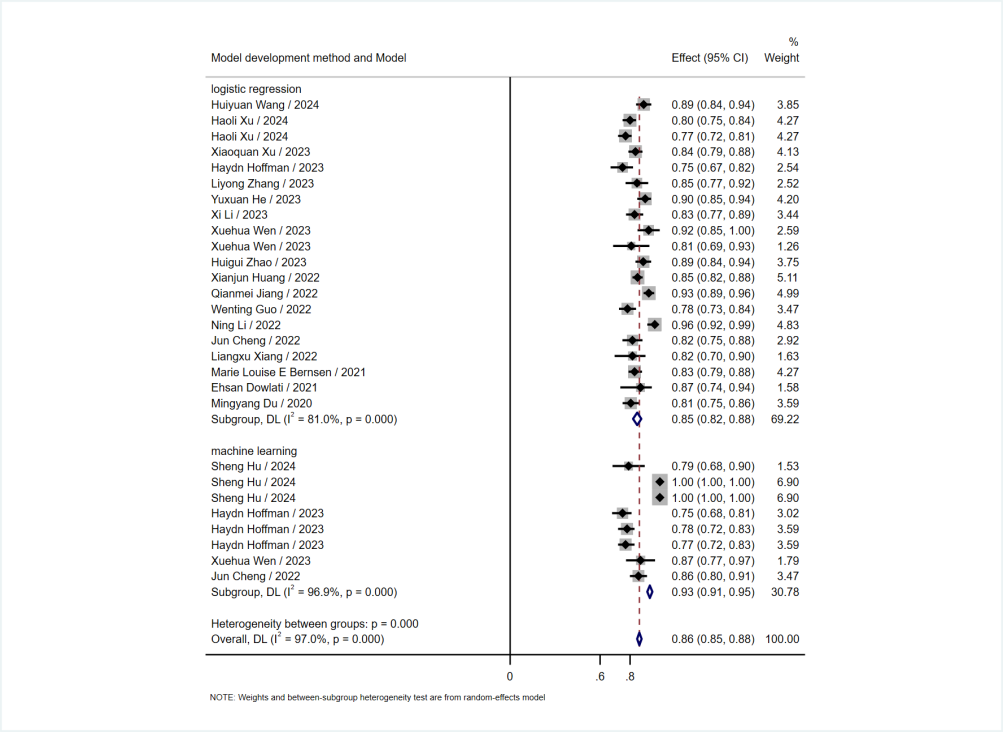


A: subgroup analysis based on the participant; B: subgroup analysis based on the outcome; C: subgroup analysis based on the time range; D: subgroup analysis based on the model development method; ACLVOS: anterior circulation large vessel occlusion stroke; MCE: malignant cerebral edema; mMCAI: malignant middle cerebral artery infarction.

Figure. 1 The AUC meta-analysis and subgroup analysis of model development

1. B.


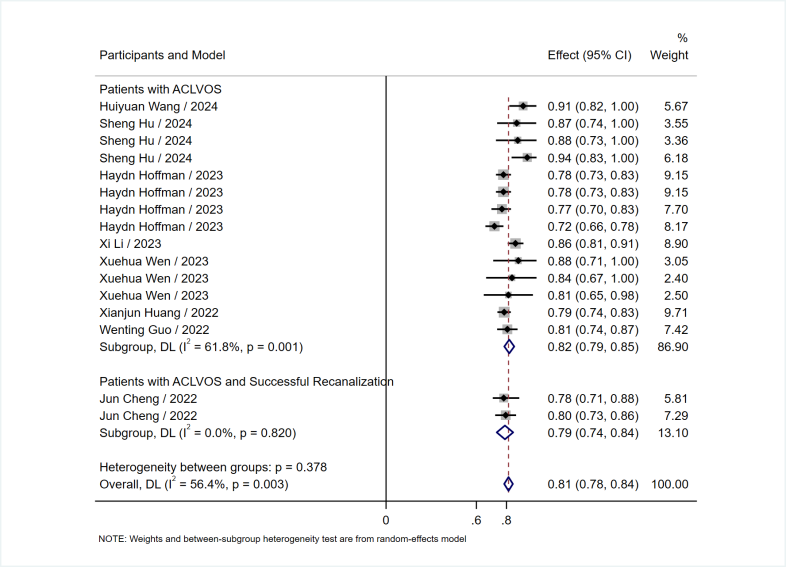

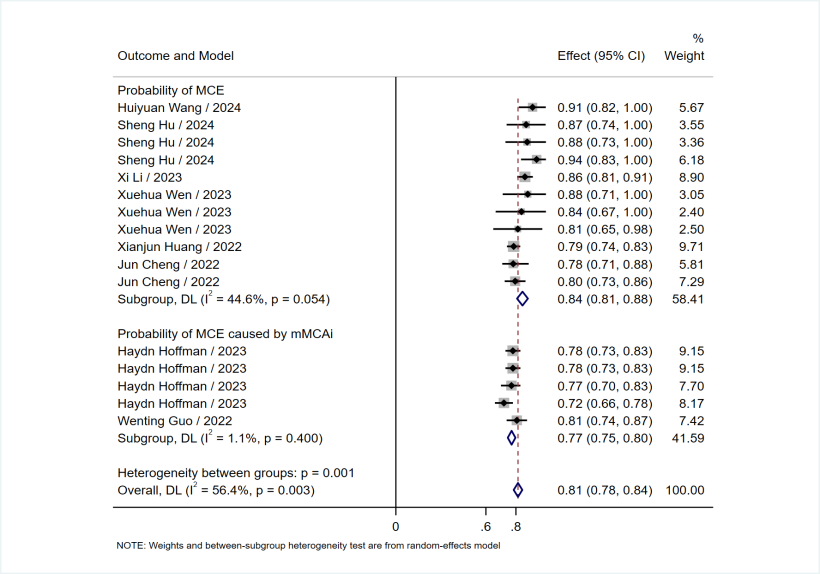


C. D.


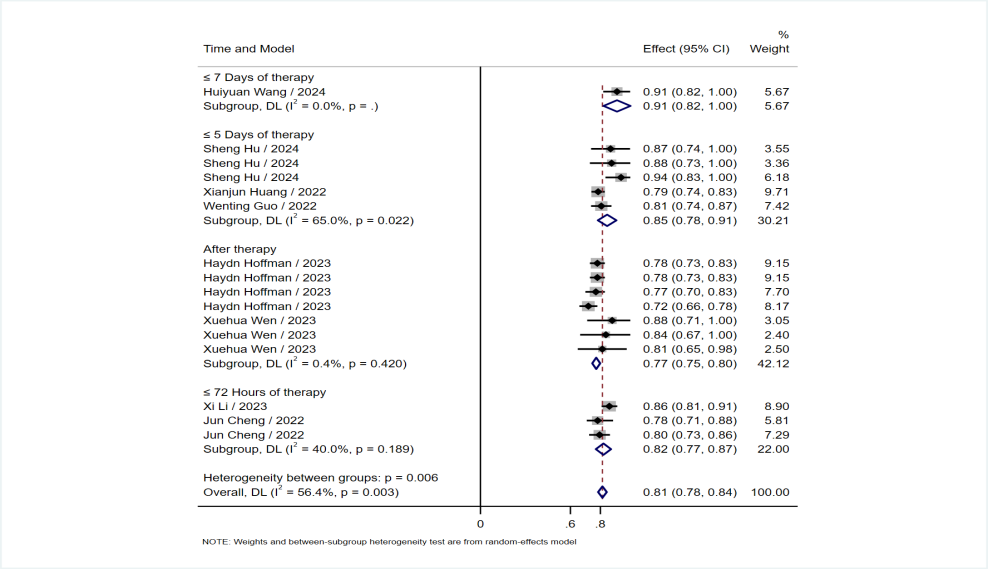

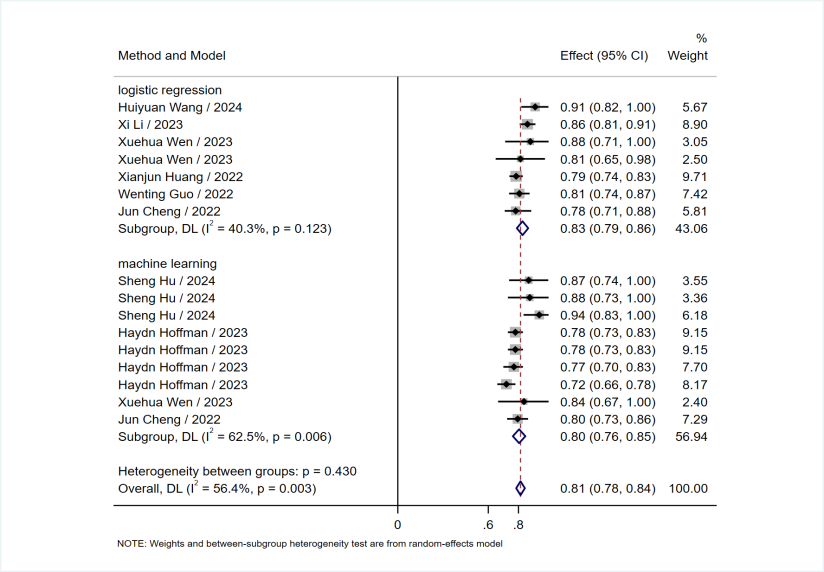


1. subgroup analysis based on the participant.
2. subgroup analysis based on the outcome.
3. subgroup analysis based on the time range.
4. subgroup analysis based on the model external validation method.

ACLVOS: anterior circulation large vessel occlusion stroke; MCE: malignant cerebral edema; mMCAI: malignant middle cerebral artery infarction.

Figure. 2 The AUC meta-analysis and subgroup analysis of model external validation
