# Supplementary material for: Risk prediction models for malignant cerebral edema after endovascular therapy in patients with acute anterior circulation large vessel occlusion stroke: a systematic review and meta-analysis
Source: Front Neurol. 2026 Feb 5;17:1686413. doi: 10.3389/fneur.2026.1686413 (PMC12916362; doi:10.3389/fneur.2026.1686413)
Supplement: Supplementary file 5 [file Table_1.DOCX]

| **Table S1**  Characteristic of of included studies (n=21) | | | | | | | | | | | |
| --- | --- | --- | --- | --- | --- | --- | --- | --- | --- | --- | --- |
| Author / Year | Country | Study Type | Study Design | Participants | Treatment | Time Range of prediction | Sample Size | | Diagnosis Criteria | MCE cases/incidence(%) | |
|  |  |  |  |  |  |  | MD | MV |  | MD | MV |
| Huiyuan Wang / 2024[28] | China | MD and MV | Cohort | ACLVOS | MT | ≤ 7 Days after MT | 247 | 185 | A, B | 59/23.89% | 28/15.14% |
| Sheng Hu / 2024[29] | China | MD and MV | Cohort | ACLVOS | MT | ≤ 5 Days after MT | 121 | 30 | B | 28/18.54% | |
| Haoli Xu / 2024[30] | China | MD | Case-control | ACLVOS and SR | MT | After MT | 398 | NR | B, C, D | 90/22.61% | NR |
| Xiaoquan Xu / 2023[31] | China | MD | Cohort | ACLVOS | MT | ≤ 7 Days after MT | 559 | NR | E, F | 74/13.25% | NR |
| Frans Kauw / 2023[32] | Netherlands | MD | Cohort | ACLVOS | ET | ≤ 7 Days after ET | 683 | NR | B, C, D, G | 40/5.86% | NR |
| Haydn Hoffman / 2023[33] | USA | MD and MV | Cohort | ACLVOS | MT | After MT | 304 | 77 | F | 50/13.12% | |
| Liyong Zhang / 2023[34] | China | MD | Case-control | ACLVOS and SR | ET | ≤ 72 Hours after ET | 114 | NR | B, G | 40/35.1% | NR |
| Jun Tong / 2023[35] | China | MD and MV | Case-control | ACLVOS | ET | ≤ 72 Hours after ET | 90 | 38 | A, B, G | 48/53.33% | 16/42.11% |
| Yuxuan He / 2023[36] | China | MD | Case-control | ACLVOS | MT | After MT | 381 | NR | B | 66/17.32% | NR |
| Xi Li / 2023[37] | China | MD and MV | Case-control | ACLVOS | MT | ≤ 72 Hours after MT | 218 | 94 | B, G | 50/22.94% | NR |
| Xuehua Wen / 2023[38] | China | MD and MV | Case-control | ACLVOS | ET | After ET | 77 | 34 | B, C | 22/28.57% | 10/29.41% |
| Huigui Zhao / 2023[39] | China | MD | Case-control | ACLVOS | MT | After MT | 159 | NR | B, C | 40/25.2% | NR |
| Xianjun Huang / 2022[40] | China | MD and MV | Cohort | ACLVOS | MT | ≤ 5 Days after MT | 643 | 773 | A, B | 135/21% | 187/24.19% |

| Author / Year | Country | Study Type | Study Design | Participants | Treatment | Time Range of prediction | Sample Size | | Diagnosis Criteria | MBE cases/incidence(%) | |
| --- | --- | --- | --- | --- | --- | --- | --- | --- | --- | --- | --- |
|  |  |  |  |  |  |  | MD | MV |  | MD | MV |
| Qianmei Jiang / 2022[41] | China | MD and MV | Case-control | ACLVOS | MT | ≤ 5 Days after MT | 329 | NR | B | 72/21.88% | NR |
| Wenting Guo / 2022[42] | China | MD and MV | Cohort | ACLVOS | MT | ≤ 5 Days after MT | 372 | 247 | F, H | 81/21.77% | 47/19.03% |
| Ling Li / 2022[43] | China | MD and MV | Case-control | ACLVOS | ET | After ET | 120 | NR | F, I | 24/20% | NR |
| Jun Cheng / 2022[44] | China | MD and MV | Case-control | ACLVOS and SR | ET | ≤ 72 Hours after ET | 267 | 115 | A, B, J | 41/15.36% | NR |
| Liangxu Xiang / 2022[45] | China | MD | Case-control | ACLVOS and SR | MT | After MT | 62 | NR | B, G | 23/37.10% | NR |
| Marie Louise E Bernsen / 2021[46] | Netherlands | MD | Cohort | ACLVOS | ET | ≤ 7 Days after ET | 1445 | NR | B, C, D, G | 82/5.67% | NR |
| Ehsan Dowlati / 2021[47] | USA | MD | Case-control | ACLVOS | MT | ≤ 72 Hours after MT | 284 | NR | A, B | 64/22.54% | NR |
| Mingyang Du / 2020[48] | China | MD and MV | Case-control | ACLVOS | MT | ≤ 5 Days after MT | 370 | NR | B, C | 71/19.19% | NR |

MD: model development; MV: model validation; MCE: malignant cerebral edema; ACLVOS: anterior circulation large vessel occlusion stroke; MT: mechanical thrombectomy; A: at least 50% low-density in the middle cerebral artery territory with local brain edema; B: at leacraniectomy; D: death; E: NIHSS score at least 18 points and a decreased level of consciousness (item 1-a at least 1 point); F: at least 2/3 of middle cerebral artery (MCA) territory infarction, with compression of ventricles or midline shift; ET: endovascular therapy; G: clinical features of malignant edema, such as decreased consciousness, unilateral dilated pupil, severe neurological deficit, at least 2 points increase in NIHSS score; H: NIHSS at least 15 points and a decreased level of conscist 5 mm midline shift (MLS) of the pineal gland or septum pellucidum on computed tomography(CT) or magnetic resonance imaging(MRI); SR: successful recanalization; NR: not reported; C: need for decompressive hemiousness (item 1-a at least 1 point); I: NIHSS score at least 15 points when non-dominant hemisphere infarction or at least 20 points when dominant hemisphere infarction.And neurological deficit and disturbance of consciousness were progressively aggravated; J: an increase in the NIHSS score at least 2 points or an a decreased level of consciousness (item 1-a at least 1 point); K: imaging evidence of brain swelling, such as midline shift of brain structure or efacement of basal cisterns.
