# Supplementary material for: Risk prediction models for malignant cerebral edema after endovascular therapy in patients with acute anterior circulation large vessel occlusion stroke: a systematic review and meta-analysis
Source: Front Neurol. 2026 Feb 5;17:1686413. doi: 10.3389/fneur.2026.1686413 (PMC12916362; doi:10.3389/fneur.2026.1686413)
Supplement: Supplementary file 6 [file Table_2.DOCX]

**Table S2** Characteristic of included prediction models (n=21)

| Author / Year | Missing data  handling | Number of variable to be selected | Variable selection method | Continuous variable  processing method | Model presentation | Model development  method | Model validation | | Model performance | | Risk predictors |
| --- | --- | --- | --- | --- | --- | --- | --- | --- | --- | --- | --- |
|  |  |  |  |  |  |  | Internal | External | AUC/C index | Calibration  method |  |
| Huiyuan Wang / 2024[28] | exclude | 35 | Multivariate analysis | Continuous variable | nomogram | LR | Y | Y | A: 0.891  B: 0.849  C: 0.911 | H-L test | 5: Baseline CIV, Baseline CI, Baseline CBS, Baseline Neutrophil, Number of retrieval attempts |
| Sheng Hu / 2024[29] | exclude | 17 | LASSO, 10-fold cross validation, Multivariate analysis | Continuous and Categorical variable | nomogram | XGBoost machine learning | NR | Y | A: 0.790-0.999  C: 0.868-0.938 | NR | 3: HU_max_ ≥90, Baseline ASPECTS score, 22 Radiomics characteristics |
| Haoli Xu / 2024[30] | exclude | 16 | Multivariate analysis | Continuous variable | NR | LR | NR | NR | A: 0.800 | H-L test | 4: Baseline NIHSS score, Baseline ASPECTS score, CS-1 score, Baseline wNWU |
| Xiaoquan Xu / 2023[31] | multiple imputation | 36 | Multivariate analysis | Continuous variable | NR | LR | NR | NR | A: 0.836 | H-L test | 6: eTICI score, Baseline Serum glucose, Baseline ASPECTS score, Baseline CBS, Baseline CS, Pass number of thrombectomy device |
| Frans Kauw / 2023[32] | multiple imputation | 29 | Multivariate analysis | Continuous variable | NR | LR | NR | NR | A: 0.876 | NR | 8: Age, Baseline NIHSS score, Baseline ASPECTS score, ICA occlusion, CS-1 score, Onset to groin, eTICI score, CSF volume/ICV ratio |

| Author / Year | Missing data  handling | Number of variable to be selected | Variable  selection  method | Continuous variable  processing method | Model presentation | Model development  method | Model validation | | Model performance | | Risk predictors |
| --- | --- | --- | --- | --- | --- | --- | --- | --- | --- | --- | --- |
|  |  |  |  |  |  |  | Internal | External | AUC/C index | Calibration  method |  |
| Haydn Hoffman / 2023[33] | multiple imputation, Mode imputation | 29 | variance thresholding, Recursive feature elimination | Continuous variable | NR | RF machine learning | Y | Y | A: 0.750-0.780  B: 0.740-0.840  C: 0.770-0.780 | NR | 10: Age, Wake-up stroke, History of Atrial fibrillation, Occlusion site, Time to arrival, Body mass index, CS-1 score, MAP on arrival, Sex, Time to reperfusion |
| Liyong Zhang / 2023[34] | NR | 26 | Multivariate analysis | Continuous variable | nomogram | LR | NR | NR | A: 0.846 | H-L test | 4: Baseline NIHSS score, Baseline NLR, CS-2 score, Non first pass effect |
| Jun Tong / 2023[35] | NR | 14 | LASSO, 10-fold cross-validation,  Multivariate analysis | Continuous variable | nomogram | LR Classifier, machine learning | NR | Y | A: 0.959  C: 0.889 | NR | 5: Age, Baseline NIHSS score, CIV, Tmax＞6S volume, 10 radiomics characteristics |
| Yuxuan He / 2023[36] | exclude | 34 | Multivariate analysis | Continuous and Categorical variable | nomogram | LR | NR | NR | A:0.901 | H-L test | 7: Baseline hematocrit level, Baseline ASPECTS score, Right hemisphere, Baseline serum glucose, CS-2 score, Baseline NIHSS score, mTICI score |
| Xi Li / 2023[37] | NR | 18 | LASSO, Multivariate analysis | Continuous variable | nomogram | LR | NR | Y | A: 0.830  C: 0.860 | Calibration diagram | 4: History of hypertension, Time to reperfusion, CS-2 score, mTICI score |
| Xuehua Wen / 2023[38] | exclude | 22 | LASSO, 10-fold cross-validation,  Multivariate analysis | Continuous variable | NR | LR | NR | Y | A: 0.870-0.924  C: 0.813-0.879 | N | 5: 15 Radiomics characteristics, Baseline ASPECTS score, Baseline CIV, DT >3 s lesion volume, IV tPA before ET |

| Author / Year | Missing data  handling | Number of variables to be selected | Variable selection method | Continuous variable  processing method | Model presentation | Model development  method | Model validation | | Model Performance | | Risk predictors |
| --- | --- | --- | --- | --- | --- | --- | --- | --- | --- | --- | --- |
|  |  |  |  |  |  |  | Internal | External | AUC/C index | Calibration  method |  |
| Huigui Zhao / 2023[39] | exclude | 24 | Multivariate analysis | Continuous variable | Logistic regression equation, scale | LR | NR | NR | A: 0.888 | H-L test | 6::Baseline NIHSS score, Baseline NLR, Baseline CIV, Time to reperfusion, CS-2 score, postoperative HT |
| Xianjun Huang / 2022[40] | multiple imputation | 22 | Multivariate analysis | Continuous variable | Logistic regression equation, scale | LR | Y | Y | A: 0.850  B: 0.874  C: 0.785 | H-L test | 8: Baseline ASPECT score, CS-1 score, Fast blood glucose level, History of hypertension, IV tPA before MT, mTICI score, Baseline NIHSS score, ICA occlusion |
| Qianmei Jiang / 2022[41] | exclude | 52 | LASSO,  10-fold cross-validation,  Multivariate analysis | Continuous variable | nomogram | LR | Y | NR | A: 0.925  B: 0.915 | H-L test | 5: Baseline NIHSS score, TOAST etiology, brain atrophy (evaluated by GCA scale), Basal cistern effacement (postoperative CT after 24 hours), Brain parenchymal hypoattenuation area (postoperative CT after 24 hours) |
| Wenting Guo / 2022[42] | exclude | 24 | Multivariate analysis | Continuous and Categorical variable | nomogram | LR | NR | Y | A: 0.783  C: 0.806 | H-L test | 6: Baseline NIHSS score, Baseline ASPECT score, Baseline SBP, ICA occlusion, TOP, mTICI score |
| Ning Li / 2022[43] | exclude | 18 | LASSO, 10-fold cross-validation,  Multivariate analysis | Continuous variable | nomogram | LR | Y | NR | A: 0.965  B: 0.965 | H-L test | 3: mTICI score, CS-2 score, Baseline CIV |

| Author / Year | Missing data  handling | Number of variables to be selected | Variable selection method | Continuous variable  processing method | Model presentation | Model development  method | Model validation | | Model Performance | | Risk predictors |
| --- | --- | --- | --- | --- | --- | --- | --- | --- | --- | --- | --- |
|  |  |  |  |  |  |  | Internal | External | AUC/C index | Calibration  method |  |
| Jun Cheng / 2022[44] | exclude | 21 | Multivariate analysis | Continuous variable | NR | LR, XGBoost algorithm | NR | Y | A: 0.816-0.856  C: 0.782-0.795 | H-L test | 5: Age, CS-2 score, Baseline NIHSS score, Time to reperfusion, Number of retrieval attempts |
| Liangxu Xiang / 2022[45] | exclude | 18 | Multivariate analysis | Continuous variable | NR | LR | NR | NR | A: 0.816 | NR | 2: NLR, FAR |
| Marie Louise E Bernsen / 2021[46] | multiple imputation | 24 | Multivariate analysis | Continuous variable | NR | LR | NR | NR | A: 0.830 | H-L test | 7: Age, Baseline NIHSS score, Baseline ASPECT score, ICA/ICA-T occlusion, CS-1 score, TOP, eTICI score |
| Ehsan Dowlati / 2021[47] | NR | 21 | Multivariate analysis | Continuous variable | NR | LR | NR | NR | A: 0.870 | NR | 2: postoperative HT, NPi |
| Mingyang Du / 2020[48] | exclude | 24 | Multivariate analysis | Continuous variable | nomogram | LR | Y | NR | A: 0.805  B: 0.805 | H-L test | 5: Age, Baseline NIHSS score, Fast blood glucose, mTICI score, CS-3 score |

AUC: area under the curve; LR: logistic regression; Y: Yes; A: model development; B: internal validation; C: external validation; H-L: Hosmer-Lemeshow; DCA: decision curve analysis; CIV: core infarct volumes; CI: collateral index; CBS: clot burden Score; LASSO: least absolute shrinkage and selection operator; XGBoost: extreme gradient boosting; NR: not reported; HU_max_: the maximum Hounsfield unit; ASPECTS: Alberta Stroke Program Early Computed Tomography Score; NIHSS: National Institutes of Health Stroke Scale; CS: collateral score; CS-1: according to Tan's standard, collateral was scored on a scale of 0-3 by percentage of collateral filling; wNWU: weighted net water uptake; eTICI: extended Thrombolysis In Cerebral Infarction; ICA: internal carotid artery; CSF: cerebrospinal fluid; ICV: intracranial volume; RF: random forest; MAP: mean arterial pressure; NLR: the ratio of neutrophil count to lymphocyte count; CS-2: according to standard of American Society of Interventional and Therapeutic Neuroradiology/Society of Interventional Radiology (ASITN/SIR), collateral was scored on a scale of 0-4 and divided into poor collateral circulation (0-2) with good collateral circulation (3-4); T_max_: time to the maximum of the residue function; mTICI: modified Thrombolysis in Cerebral Infarction; DT: delay time; IV tPA: intravenous tissue type plasminogen activator; ET: Endovascular Treatment; HT: hemorrhagic transformation; MT: mechanical Thrombectomy; TOAST: Trial of Org 10712 in Acute Stroke; GCA: Global Cortical Atrophy; CT: computed tomography; SBP: systolic blood pressure; TOP: time to puncture; FAR: the ratio of fibrinogen level to albumin level; ICA-T: internal carotid artery terminus; NPi: neurological pupil index; CS-3: collateral was scored on a scale of 0-3 by reconstitution of occluded territory.
