# Supplementary material for: Risk prediction models for malignant cerebral edema after endovascular therapy in patients with acute anterior circulation large vessel occlusion stroke: a systematic review and meta-analysis
Source: Front Neurol. 2026 Feb 5;17:1686413. doi: 10.3389/fneur.2026.1686413 (PMC12916362; doi:10.3389/fneur.2026.1686413)
Supplement: Supplementary file 7 [file Table_3.DOCX]

| **Table S3.** results of quality assessment (n=21) | | | | | | | | | | | |
| --- | --- | --- | --- | --- | --- | --- | --- | --- | --- | --- | --- |
| Study | ROB | | | |  | Applicability | | |  | Overall | |
|  | Participants | Predictors | Outcome | Analysis |  | Participants | Predictors | Outcome |  | ROB | Applicability |
| Huiyuan Wang / 2024 | + | - | + | - |  | + | - | + |  | - | - |
| Sheng Hu / 2024 | + | ？ | + | - |  | + | - | + |  | - | - |
| Haoli Xu / 2024 | - | - | - | - |  | - | - | + |  | - | - |
| Xiaoquan Xu / 2023 | + | + | + | - |  | + | + | - |  | - | - |
| Frans Kauw / 2023 | + | + | + | - |  | + | - | + |  | - | - |
| Haydn Hoffman / 2023 | + | - | - | - |  | + | - | - |  | - | - |
| Liyong Zhang / 2023 | - | - | - | - |  | - | + | + |  | - | - |
| Jun Tong / 2023 | - | - | + | - |  | + | - | + |  | - | - |
| Yuxuan He / 2023 | - | - | - | - |  | + | + | + |  | - | + |
| Xi Li / 2023 | - | ？ | - | - |  | + | + | + |  | - | + |
| Xuehua Wen / 2023 | - | - | - | - |  | + | - | + |  | - | - |
| Huigui Zhao / 2023 | - | - | - | - |  | + | + | + |  | - | + |
| Xianjun Huang / 2022 | + | ? | + | - |  | + | + | + |  | - | + |
| Qianmei Jiang / 2022 | - | - | + | - |  | + | - | + |  | - | - |
| Wenting Guo / 2022 | + | ? | ? | - |  | + | + | - |  | - | - |
| Ning Li / 2022 | - | - | - | - |  | + | + | - |  | - | - |
| Jun Cheng / 2022 | - | - | - | - |  | - | + | + |  | - | - |
| Liangxu Xiang / 2022 | - | - | - | - |  | - | + | + |  | - | - |
| Marie Louise E Bernsen / 2021 | + | + | + | - |  | + | + | - |  | - | - |
| Ehsan Dowlati / 2021 | - | - | - | - |  | + | - | - |  | - | - |
| Mingyang Du / 2020 | - | - | + | - |  | + | + | + |  | - | + |

ROB: risk of bias; +: indicates low ROB/low concern regarding applicability; -: indicates high ROB/high concern regarding applicabiity; ?: indicates unclear ROB/unclear concern regarding applicability.
