# Supplementary material for: Risk prediction models for malignant cerebral edema after endovascular therapy in patients with acute anterior circulation large vessel occlusion stroke: a systematic review and meta-analysis
Source: Front Neurol. 2026 Feb 5;17:1686413. doi: 10.3389/fneur.2026.1686413 (PMC12916362; doi:10.3389/fneur.2026.1686413)
Supplement: Supplementary file 8 [file Table_4.DOCX]

**Table S4.** meta-analysis and subgroup analysis of AUC/C index (n=21)

| Subgroup | | AUC/C index | 95% CI- | 95% CI+ |
| --- | --- | --- | --- | --- |
| Model development | | 0.86 | 0.85 | 0.88 |
| Participants | |  |  |  |
|  | AIS patients due to LVO and Successful Recanalization | 0.81 | 0.78 | 0.84 |
|  | AIS patients due to LVO | 0.88 | 0.86 | 0.89 |
| Prediction Outcome | |  |  |  |
|  | Probability of MCE caused by mMCAi | 0.81 | 0.75 | 0.87 |
|  | Probability of MCE | 0.89 | 0.87 | 0.90 |
| Time Range | |  |  |  |
|  | After therapy | 0.83 | 0.79 | 0.88 |
|  | ≤ 72 Hours of therapy | 0.84 | 0.81 | 0.86 |
|  | ≤ 5 Days of therapy | 0.94 | 0.93 | 0.96 |
|  | ≤ 7 Days of therapy | 0.86 | 0.81 | 0.92 |
| Model development method | |  |  |  |
|  | machine learning | 0.93 | 0.91 | 0.95 |
|  | logistic regression | 0.85 | 0.82 | 0.88 |
| External validation | | 0.81 | 0.78 | 0.84 |
| Participants | |  |  |  |
|  | AIS patients due to LVO and Successful Recanalization | 0.79 | 0.74 | 0.84 |
|  | AIS patients due to LVO | 0.82 | 0.79 | 0.85 |
| Prediction Outcome | |  |  |  |
|  | Probability of MCE caused by mMCAi | 0.77 | 0.75 | 0.80 |
|  | Probability of MCE | 0.84 | 0.81 | 0.88 |
| Time Range | |  |  |  |
|  | After therapy | 0.77 | 0.75 | 0.80 |
|  | ≤ 72 Hours of therapy | 0.82 | 0.77 | 0.87 |
|  | ≤ 5 Days of therapy | 0.85 | 0.78 | 0.91 |
|  | ≤ 7 Days of therapy | 0.91 | 0.82 | 1.00 |
| Model external validation method | |  |  |  |
|  | machine learning | 0.83 | 0.79 | 0.86 |
|  | logistic regression | 0.80 | 0.76 | 0.85 |

AUC: Reported area under the curve; AIS: acute ischemic stroke; LVO: large vessel occlusion; MCE: malignant cerebral edema; mMCAi: malignant middle cerebral artery infarction
